# Association between serum uric acid levels and atrial fibrillation in different fasting glucose patterns: A case-control study

**DOI:** 10.3389/fendo.2023.1021267

**Published:** 2023-01-23

**Authors:** Xia Zhong, Huachen Jiao, Dongsheng Zhao, Mengqi Yang, Jing Teng

**Affiliations:** ^1^Department of First Clinical Medical College, Shandong University of Traditional Chinese Medicine, Jinan, Shandong, China; ^2^Department of Cardiology, Affiliated Hospital of Shandong University of Traditional Chinese Medicine, Jinan, Shandong, China

**Keywords:** atrial fibrillation, serum uric acid, glucose metabolism, inflammation, diabetes mellitus

## Abstract

**Background:**

Previous studies have shown both dysglycaemia and hyperuricemia are associated with an increased risk of atrial fibrillation (AF), while the relationship between serum uric acid (SUA) levels and AF in different fasting glucose patterns (FBG) is unclear. Therefore, this study aimed to determine the association between SUA and AF in different FBG patterns.

**Methods:**

A total of 1840 patients in this case-control study were enrolled, including 920 AF patients and 920 controls. Patients were divided into three groups according to the different FBG patterns: normoglycemic, impaired fasting glucose (IFG), and diabetes mellitus (DM). Multivariate logistic regression models were performed to evaluate the relationship between SUA and AF in different FBG patterns. Pearson correlation analysis was used to explore the correlation between SUA and metabolic factors. Receiver operating characteristic (ROC) curve models indicated the diagnostic efficiency of SUA for diagnosing AF.

**Results:**

SUA was independently associated with AF after adjusting for all confounding factors in different FBG patterns(normoglycemic: OR=1.313, 95% CI:1.120-1.539; IFG: OR=1.386, 95% CI:1.011-1.898; DM: OR=1.505, 95% CI:1.150-1.970). Pearson’s correlation analysis suggested that SUA in AF patients was correlated with several different metabolic factors in different FBG patterns (p<0.05). ROC curve analysis showed that SUA in the normoglycemic group combined with CHD and APOB [AUC: 0.906 (95% CI: 0.888-0.923)], in the IFG group combined with CHD and Scr [AUC: 0.863 (95% CI: 0.820-0.907)], in the DM group combined with CHD and SBP [AUC: 0.858 (95% CI: 0.818-0.898)] had the highest AUC for predicting AF.

**Conclusion:**

Findings implied a significant association between SUA and AF in different FBG patterns and provide specific models combined with other factors (CHD, APOB, SCr, SBP), which might contribute to the diagnosis of AF.

## Introduction

With the life expectancy increasing, the dramatic rise in prevalence and incidence of atrial fibrillation(AF) is emerging as an urgent public health concern worldwide (1, 2). Currently, AF is affecting about 33.5 million individuals involving 2.5-3.5% of the population in several countries (3). It is estimated that the global prevalence of AF will increase by more than 60% by 2050, and the prevalence of AF in China will be ~2.3-fold higher than the equivalent predicted in the United States (1, 4). Although the pathophysiology of AF is not well understood, it is increasingly recognized that inflammation and oxidative stress have been recognized as potential essential mechanisms for the onset and maintenance of AF. When associating inflammation and oxidative stress with the development of AF, it is significant to mention the culprit of cardiovascular and non-cardiovascular outcomes, including myocardial infarction, heart failure, stroke, cognitive decline, as well as a higher risk of all-cause mortality in this phenomenon (5). Despite multifaceted efforts, the management of the AF population remains a concern. Recently, serum biomarkers are emerging as popular indices increasingly showing potential value in AF risk stratification and adjunctive treatment decisions.

Hyperuricemia is a metabolic disease caused by a disturbance of purine metabolism or uric acid excretion, which increases the risk of cardiovascular disease through various pathways. As reported previously, increased serum uric acid (SUA) levels may contribute to the development of AF through the activation of xanthine oxidoreductase (XOR) and the activation of the NLRP3 inflammasome induced by monosodium urate (MSU) crystals (6), meanwhile, it is also associated with vasoconstriction, endothelial dysfunction, and insulin resistance (7). In recent years, numerous studies have established the correlation between elevated SUA and AF, and hyperuricemia has also been recognized as an independent competing risk factor for AF (8). Several meta-analyses indicated that elevated SUA is associated with an increased risk of AF (9–11). Several other studies have reported contradictory sex associations between elevated SUA and AF (12–16).

Moreover, studies have shown that elevated SUA contributes to cardiovascular disease, and might be associated with abnormal lipid and glucose metabolism (17). In detail, hyperuricemia can trigger abnormalities in glucose metabolism, such as hyperinsulinemia or diabetes (DM) status; and impaired renal function due to abnormal glucose metabolism ultimately induces hyperuricemia (18). Nevertheless, very little information is currently available regarding the relationship between SUA and AF in patients with T2DM; in particular, there is no evidence to determine whether the association between SUA and AF is consistent in different FBG metabolism patterns. Therefore, we conducted this case-control study based on Chinese adults to evaluate the relationship between SUA and AF under different FBG metabolism conditions.

## Materials and methods

### Study design and data source

All data involved in this case-control study are based on the electronic medical record database of the Affiliated Hospital of Shandong University of Traditional Chinese Medicine. This database contains anonymously obtained clinical, demographic, and medication information, and several studies focusing on assessing the relationship between serum biomarkers and AF have already been conducted based on this database (19–22). We reviewed clinical information from 920 patients with AF who were diagnosed by specialized cardiologists and required hospitalization between January 2019 and September 2021, who were diagnosed on admission and were admitted for systemic care for an episode of AF. Meanwhile, we matched patients with sinus rhythm and non-atrial fibrillation collected during the same time period in a 1:1 ratio, and a total of 1840 patients were enrolled, including 920 patients with AF and 920 age- and sex-matched (1:1) non-AF patients with sinus rhythm. Patients with AF were identified as having a prolonged duration of arrhythmia, with a 12-lead ECG recorded or lasting at least 30 seconds (23). Inclusion criteria were as follows: 1) aged 28-85 years; 2) complete medical information. Exclusion criteria were as follows: 1) congenital heart disease, valvular disease, heart failure, or cardiac surgery; 2) severe infection, malignant tumor, or autoimmune disease; 3) severely impaired liver function, or hyperthyroidism; 4) impaired renal function; patients with estimated glomerular filtration rate(eGFR)<60 mL/(min·1.73m^2^) and clinical symptoms were diagnosed with impaired renal function when assessed by their professional physicians; 5) currently undergoing treatment anticoagulants, diuretics and lipid-lowering drugs other than statins that may affect blood lipid levels; 6) patients who received uric acid lowering drugs and antidiabetic drugs because of drug intolerance or refusal; 7) pregnant or lactating women. The study This study was approved by the ethical committee of the Affiliated Hospital of Shandong University of Traditional Chinese Medicine (NO.20200512FA62) and the informed consent was waived due to data being anonymized.

### Study variables

We reviewed all patient medical data including demographic variables and clinical variables based on an electronic medical record database. Age, gender, systolic blood pressure (SBP), diastolic blood pressure (DBP); comorbidities, medication, and laboratory parameters were collected and included. Specifically, comorbidities included hypertension and coronary heart disease(CHD); medication information included β-blockers, CCBs, ACEI/ARB, and statins;

laboratory indicators included serum uric acid (SUA), fasting blood glucose (FBG), triglycerides (TG), cholesterol (TC), low-density lipoprotein cholesterol (LDL-C), high-density lipoprotein cholesterol (HDL-C), apolipoprotein A1 (APOA1), apolipoprotein B (APOB), lipoprotein (a) [Lp (a)], aspartate aminotransferase (AST); alanine aminotransferase (ALT); serum creatinine (SCr), and albumin (ALB). Hyperuricemia was diagnosed if SUA > 7.0 mg/dL in men or > 6.0 mg/dL in women (24). In addition, patients were divided into three groups according to the different FBG patterns (25): normoglycemic group (FBG < 6.1 mmol/L), impaired fasting glucose (IFG) group (6.1–7.0 mmol/L), and diabetes mellitus (DM) group (FBG ≥ 7.0 mmol/L or receiving hypoglycemic therapy).

### Statistical analysis

Statistical analysis was performed by SPSS version 26.0, GraphPad Prism version 9.0.0, and python version 3.6.8. Quantitative data were expressed as means ± standard deviations(SD) or medians (interquartile ranges), and differences between groups were compared using the T-test or the Mann-Whitney U test. Categorical variables were described as frequencies (percentages)and chi-square tests were applied to analyze differences between groups. Pearson correlation analysis was used to assess the correlation between SUA and several metabolic factors in different FBG patterns. Taking AF as the dependent variable, multivariate logistic regression models were used to adjust for confounding factors and showed the relationship between SUA and AF in different FBG groups. The receiver operating characteristic (ROC) curve model revealed the diagnostic efficiency of SUA combined with related indicators for diagnosing AF in different FBG states. A p < 0.05 was considered to be significant, and two-tailed.

## Results

### Baseline characteristics of the individuals

**Table 1** shows the baseline characteristics of the AF and control populations in different fasting glucose patterns, including 1203 normoglycemic patients, 283 patients with impaired fasting glucose, and 354 patients with diabetes. Compared with the controls, AF patients were more likely to experience CHD and hypertension and use β-blockers, CCBs, ACEI/ARB, and statins (all p<0.005) in different FBG metabolism patterns; meanwhile, there were significant differences under different FBG patterns in SBP, DBP, TC, LDL-C, HDL-C, APOA1, APOB, SCr, ALB, and SUA (all p < 0.05).

**Table 1:** Baseline characteristics of paroxysmal AF group and controls.

Variables	Normoglycemic group (N=1203)			IFG group (N=283)			DM group (N=354)		
	AF group (N=590)	Control group (N=613)	P value	AF group (N=138)	Control group (N=145)	P value	AF group (N=192)	Control group (N=162)	P value
Age, years	67.75 ± 11.12	66.56 ± 12.17	0.077	69.28 ± 9.19	68.94 ± 9.43	0.759	70.83 ± 8.17	73.09 ± 8.52	0.011*
Gender			0.840			0.208			0.709
Men	293 (49.66)	308 (50.24)		76 (55.07)	69 (47.59)		91 (47.40)	80 (49.38)	
Women	297 (50.34)	305 (49.76)		62 (44.93)	76 (52.41)		101 (52.60)	82 (50.62)	
CHD, n (%)	513 (86.95)	130 (21.21)	<0.001*	122 (88.41)	42 (28.97)	<0.001*	177 (92.19)	55 (33.95)	<0.001*
Hypertension, n (%)	377 (63.90)	160 (26.10)	<0.001*	100 (72.46)	60 (41.38)	<0.001*	141 (73.44)	88 (54.32)	<0.001*
SBP, mmHg	129.74 ± 18.78	132.95 ± 18.89	0.003*	131.96 ± 19.44	139.83 ± 19.72	0.001*	133.77 ± 20.81	143.00 ± 20.50	<0.001*
DBP, mmHg	77.77 ± 13.48	80.85 ± 11.69	<0.001*	79.67 ± 14.18	84.57 ± 12.91	0.003*	78.84 ± 13.63	82.38 ± 12.59	0.012*
FBG, mmol/L	5.16 ± 0.53	5.30 ± 0.44	<0.001*	6.50 ± 0.24	6.48 ± 0.24	0.484	9.27 ± 2.81	9.45 ± 2.65	0.538
TG, mmol/L	1.00[0.74-1.41]	1.06[0.79-1.45]	0.079	1.12[0.78-1.52]	1.22[0.95-1.68]	0.011*	1.19[0.91-1.68]	1.23[0.92-1.77]	0.802
TC, mmol/L	4.20 ± 1.09	5.03± 1.06	<0.001*	4.15 ± 1.13	5.19 ± 1.05	<0.001*	4.18 ± 1.07	4.88 ± 1.25	<0.001*
LDL-C, mmol/L	2.50 ± 0.91	2.97 ± 0.84	<0.001*	2.53 ± 0.84	3.13± 0.84	<0.001*	2.47 ± 0.87	2.84 ± 0.92	<0.001*
HDL-C, mmol/L	1.10 ± 0.29	1.22 ± 0.31	<0.001*	1.04 ± 0.29	1.19 ± 0.27	<0.001*	1.02 ± 0.34	1.13 ± 0.31	0.002*
APOA1, g/L	1.14 ± 0.26	1.23± 0.25	<0.001*	1.14 ± 0.26	1.24 ± 0.21	<0.001*	1.11 ± 0.29	1.19 ± 0.27	0.008*
APOB, g/L	0.77 ± 0.24	0.98± 0.23	<0.001*	0.86 ± 0.79	1.04± 0.24	0.011*	0.82 ± 0.25	0.99 ± 0.28	<0.001*
Lp (a), mg/L	14.00[6.80-30.08]	14.70[7.20-28.45]	0.814	15.15[7.23-30.93]	12.70[6.15-27.00]	0.497	14.05[6.70-30.10]	16.40[6.28-32.13]	0.550
AST, U/L	20.00[16.00-25.00]	19.00[16.00-23.00]	0.001*	20.00[16.75-27.00]	19.00[16.00-24.00]	0.088	19.00[16.00-25.00]	17.00[14.00-23.25]	0.061
ALT, U/L	15.00[12.00-22.00]	16.00[12.00-22.00]	0.412	18.50[11.00-28.00]	18.00[13.00-25.50]	0.813	17.00[12.00-24.00]	18.00[13.00-25.00]	0.381
SCr, μmoI/L	72.00[60.00-83.25]	63.00[54.00-74.00]	<0.001*	72.00[58.00-88.25]	62.00[53.00-74.00]	<0.001*	70.00[57.00-87.00]	58.00[52.00-68.00]	<0.001*
SUA, mg/dL	5.89 ± 1.72	5.12 ± 1.34	<0.001*	5.88 ± 1.80	5.22 ± 1.38	0.001*	5.87 ± 1.86	5.02 ± 1.46	<0.001*
ALB, g/L	38.11± 4.29	40.04 ± 4.05	<0.001*	38.49 ± 4.67	40.91 ± 3.62	<0.001*	37.42± 5.54	39.60 ± 4.75	<0.001*
β-blockers, n (%)	462 (78.31)	79 (12.89)	<0.001*	107 (77.54)	32 (22.07)	<0.001*	152 (79.17)	42 (25.93)	<0.001*
CCBs, n (%)	200 (33.90)	85 (13.87)	<0.001*	56 (40.58)	31 (21.38)	<0.001*	75 (39.06)	45 (27.78)	<0.001*
ACEI/ARB, n (%)	327 (55.42)	73 (11.91)	<0.001*	77 (55.80)	22 (15.17)	<0.001*	109 (56.77)	40 (24.69)	<0.001*
statins, n (%)	377 (63.90)	122 (19.90)	<0.001*	94 (68.12)	40 (27.59)	<0.001*	134 (69.79)	49 (30.25)	<0.001*

Data were presented as mean±SD, median [interquartile range], or n (%).

*Statistically significant value (P<0.05).

AF, atrial fibrillation; IFG, impaired fasting glucose; DM, diabetes mellitus; CHD, coronary heart disease; SBP, systolic blood pressure; DBP, diastolic blood pressure; FBG, fasting blood glucose; TG, triglycerides; TC, cholesterol; LDL-C, low-density lipoprotein cholesterol; HDL-C, high-density lipoprotein cholesterol; APOA1, apolipoprotein A1; APOB, apolipoprotein B; Lp (a), lipoprotein (a); AST, aspartate aminotransferase; ALT, alanine aminotransferase; SCr, serum creatinine; SUA, serum uric acid; ALB, albumin.

**Figure 1:**
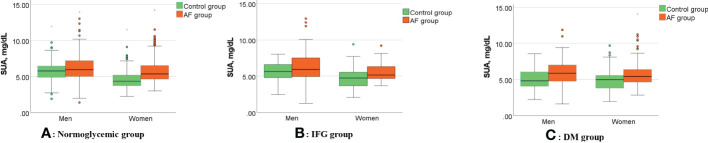
Gender differences in SUA levels between AF patients and controls under different FBG metabolic patterns. **(A–C)** Compared with controls, SUA levels of the AF patients were significantly higher in the men (all p<0.05) and women (all p<0.05) in different FBG patterns. AF, atrial fibrillation; SUA, serum uric acid; IFG, impaired fasting glucose; DM, diabetes mellitus.

**Figure 1** shows the gender differences in SUA levels between AF patients and controls under different FBG metabolic patterns. In the normoglycemic group, SUA levels in males (6.07 ± 1.87 vs. 5.69 ± 1.21 mg/dL, p=0.003, **Figure 1A**) and females (5.72 ± 1.55 vs. 4.54 ± 1.21 mg/dL, p<0.001, **Figure 1A**) with AF were significantly higher than controls. In the IFG group, SUA levels in males (6.27 ± 2.13 vs. 5.71± 1.17 mg/dL, p=0.049, **Figure 1B**) and females (5.41 ± 1.13 vs. 4.78 ± 1.42 mg/dL, p=0.005, **Figure 1B**) with AF were significantly higher than controls. In the DM group, SUA levels in males (5.87 ± 1.84 vs. 5.14 ± 1.43 mg/dL, p=0.004, **Figure 1C**) and females (5.86 ± 1.88 vs. 4.90 ± 1.49 mg/dL, p<0.001, **Figure 1C**) with AF were also significantly higher than controls.

**Figure 2:**
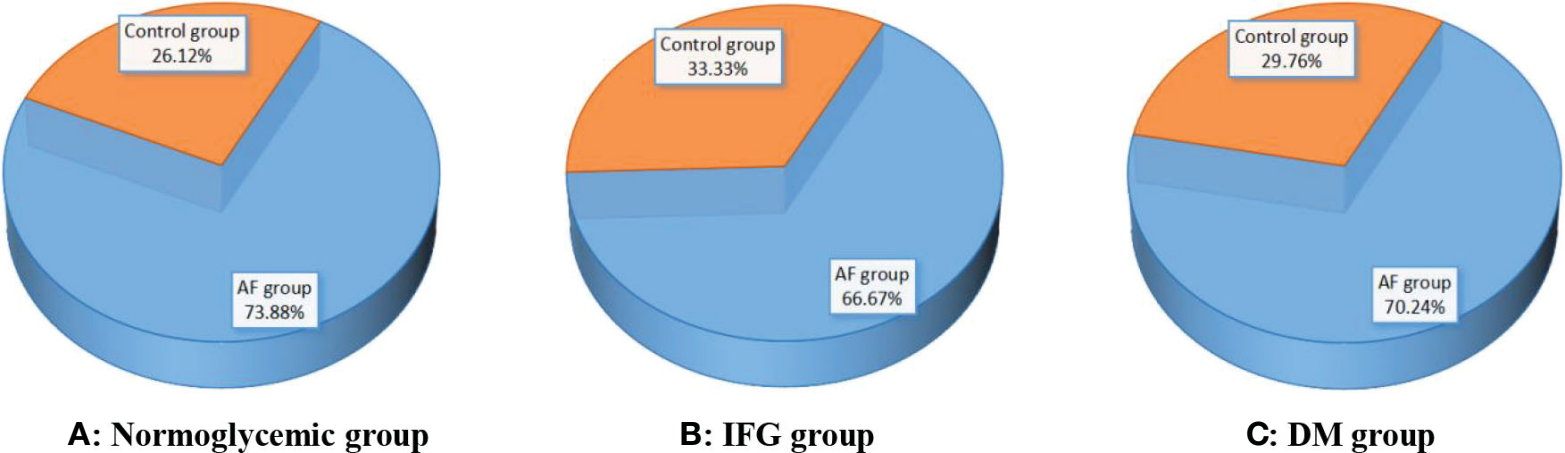
Comparison of the rate of hyperuricemia between the AF group and the control group under different FBG patterns. **(A–C)** Compared with controls, the rate of hyperuricemia in the AF group was significantly higher than those in the control group (all p < 0.001). AF, atrial fibrillation; IFG, impaired fasting glucose; DM, diabetes mellitus.

**Figure 2** shows the comparison of the rate of hyperuricemia between the AF group and the control group under different FBG patterns. In the normoglycemic group, the rate of hyperuricemia in the AF group was significantly higher than those in the control group (73.88 vs. 26.12 %, p < 0.001, **Figure 2A**). In the IFG group, the rate of hyperuricemia in the AF group was significantly higher than those in the control group (66.67 vs. 33.33 %, p < 0.001, **Figure 2B**). In the DM group, the rate of hyperuricemia in the AF group was also significantly higher than those in the control group (70.24 vs. 29.76 %, p < 0.001, **Figure 2C**).

### Multivariate logistic regression to reveal the association between SUA and AF in different FBG patterns

**Table 2** shows the relationship between SUA and AF in different FBG groups after adjusting for confounding factors. First, we found that SUA was significantly associated with AF under three FBG metabolism patterns (normoglycemic pattern: OR=1.353, 95% CI:1.195-1.531, p<0.001; IFG pattern: OR=1.378, 95% CI:1.107-1.715, p=0.004; DM pattern: OR=1.348, 95% CI:1.112-1.633, p=0.002, **Table 2**) after adjusting for age, gender, hypertension, CHD, CCBs, β-blockers, ACEI/ARB, and statins. Then, we further adjusted for TC, LDL-C, HDL-C, APOA1, APOB, SCr, and ALB, the consequence indicated that SUA still independently associated with AF in different FBG metabolism patterns (normoglycemic pattern: OR=1.451, 95% CI:1.303-1.617, p<0.001; IFG pattern: OR=1.402, 95% CI:1.101-1.786, p=0.006; DM pattern: OR=1.460, 95% CI:1.221-1.746, p<0.001, **Table 2**). Finally, we adjusted for all these factors and found that SUA remains a significantly relevant factor for AF in different FBG patterns(normoglycemic pattern: OR=1.313, 95% CI:1.120-1.539, p=0.001; IFG pattern: OR=1.386, 95% CI:1.011-1.898, p=0.042; DM pattern: OR=1.505, 95% CI:1.150-1.970, p=0.003, **Table 2**).

**Table 2:** Association between SUA and AF in different FBG patterns.

Models	Normoglycemic group (n=1203)		IFG group (N=283)		DM group (N=354)	
	OR 95% CI	P value	OR 95% CI	P value	OR 95% CI	P value
Model 1	1.407 (1.296-1.528)	<0.001*	1.308 (1.116-1.533)	0.001*	1.375 (1.193-1.584)	<0.001*
Model 2	1.353 (1.195-1.531)	<0.001*	1.378 (1.107-1.715)	0.004*	1.348 (1.112-1.633)	0.002*
Model 3	1.451 (1.303-1.617)	<0.001*	1.402 (1.101-1.786)	0.006*	1.460 (1.221-1.746)	<0.001*
Model 4	1.313 (1.120-1.539)	0.001*	1.386 (1.011-1.898)	0.042*	1.505 (1.150-1.970)	0.003*

Model 1: crude, no adjustment.

Model 2: adjusting for age, gender, hypertension, CHD, CCBs, β-blockers, ACEI/ARB, and statins.

Model 3: adjusting for TC, LDL-C, HDL-C, APOA1, APOB, SCr, and ALB.

Model 4: adjusting for all these factors.

*Statistically significant value (P<0.05).

### Correlation analysis of the SUA in AF patients with metabolic factors

**Figure 3:**
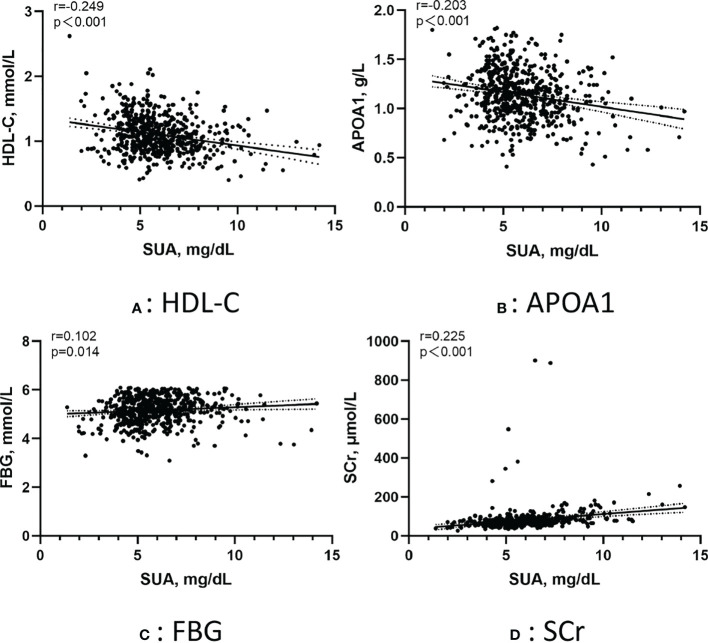
Pearson correlation of the SUA in AF patients with metabolic factors under the normoglycemic pattern. **(A)** Correlation between SUA and HDL-C; **(B)** Correlation between SUA and APOA1; **(C)** Correlation between SUA and FBG; **(D)** Correlation between SUA and SCr. SUA, serum uric acid; HDL-C, high-density lipoprotein cholesterol; APOA1, apolipoprotein A1; FBG, fasting blood glucose; SCr, serum creatinine.

**Figure 4:**
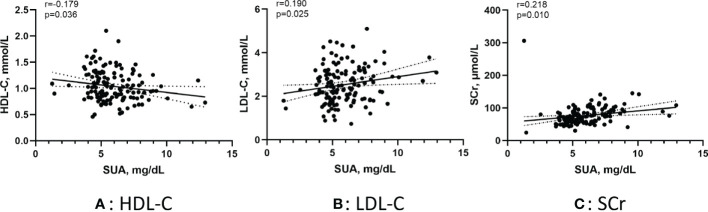
Pearson correlation of the SUA in AF patients with metabolic factors under the IFG pattern. **(A)** Correlation between SUA and HDL-C; **(B)** Correlation between SUA and LDL-C; **(C)** Correlation between SUA and SCr. SUA, serum uric acid; IFG, impaired fasting glucose; HDL-C, high-density lipoprotein cholesterol; LDL-C, low-density lipoprotein cholesterol; SCr, serum creatinine.

**Figure 3** shows the correlation between the SUA in AF patients and several metabolic factors under the normoglycemic pattern. Pearson correlation analysis suggested that HDL-C (r =-0.249, p < 0.001) and APOA1 (r =-0.203, p < 0.001) were negatively correlated with SUA,whereas FBG (r =0.102, p=0.014) and SCr (r =0.225, p < 0.001) were positively correlated with SUA. **Figure 4** shows the correlation between the SUA in AF patients and several metabolic factors under the IFG pattern. Pearson correlation analysis suggested that HDL-C (r =-0.179, p=0.036) was negatively correlated with SUA,whereas LDL-C (r =0.190, p=0.025) and SCr (r =0.218, p=0.010) were positively correlated with SUA. Interestingly, we didn’t find a correlation between SUA and metabolic factors under the DM pattern in the AF population.

### SUA combined with related indicators for diagnosing AF in different FBG patterns

**Figure 5:**
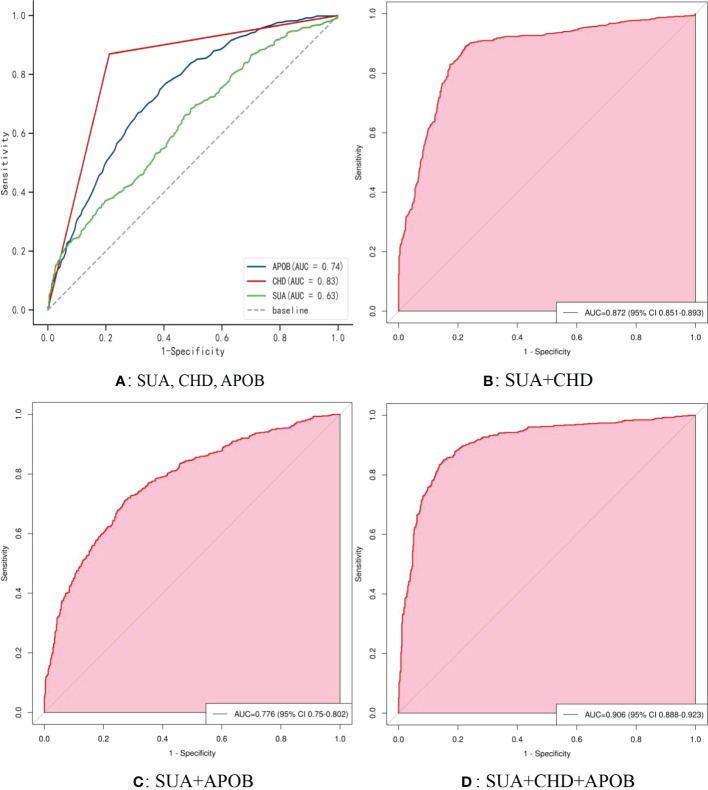
The receiver operating characteristic (ROC) curve model of SUA for diagnosing AF in the normoglycemic pattern. **(A)** Performance of SUA, CHD, and APOB for predicting AF. **(B)** Performance of SUA combined with CHD for predicting AF. **(C)** Performance of SUA combined with APOB for predicting AF. **(D)** Performance of SUA combined with CHD and APOB for predicting AF. SUA, serum uric acid; CHD, coronary heart disease; APOB, apolipoprotein B.

**Figure 6:**
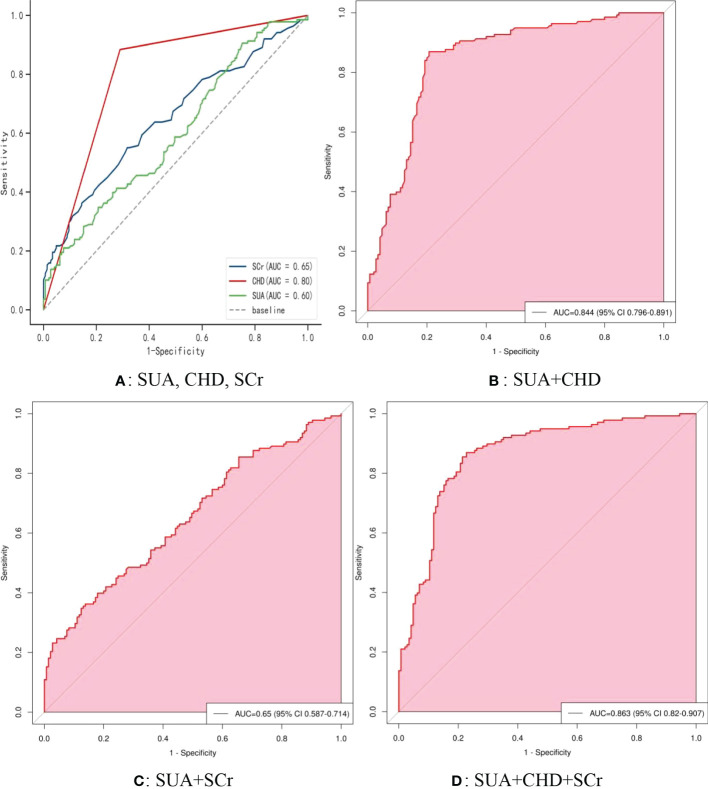
The receiver operating characteristic (ROC) curve model of SUA for diagnosing AF in the IFG pattern. **(A)** Performance of SUA, CHD, and FBG for predicting AF. **(B)** Performance of SUA combined with CHD for predicting AF. **(C)** Performance of SUA combined with FBG for predicting AF. **(D)** Performance of SUA combined with CHD and FBG for predicting AF. IFG, impaired fasting glucose; SUA, serum uric acid; CHD, coronary heart disease; FBG, fasting blood glucose.

**Figure 7:**
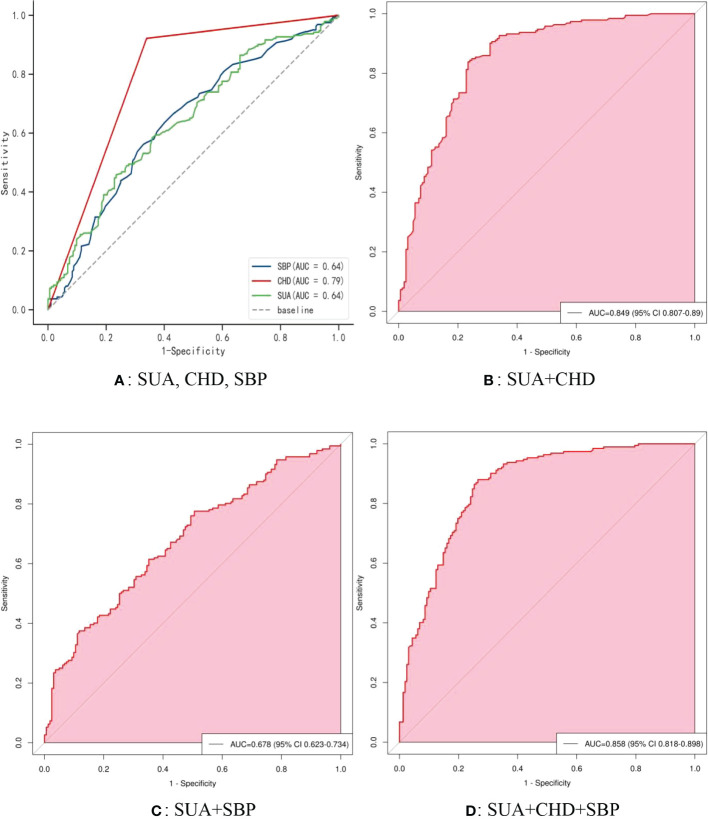
The receiver operating characteristic (ROC) curve model of SUA for diagnosing AF in the DM pattern. **(A)** Performance of SUA, CHD, and SBP for predicting AF. **(B)** Performance of SUA combined with CHD for predicting AF. **(C)** Performance of SUA combined with SBP for predicting AF. **(D)** Performance of SUA combined with CHD and SBP for predicting AF. DM, diabetes mellitus; SUA, serum uric acid; CHD, coronary heart disease; APOB, apolipoprotein B; SBP, systolic blood pressure.

**Figure 5** shows the receiver operating characteristic (ROC) curve model of SUA for diagnosing AF in the normoglycemic pattern. In the normoglycemic pattern, the ROC curve analysis suggested that the AUCs for taking SUA, CHD, and APOB to predict AF were 0.63, 0.83, and 0.74, respectively (**Figure 5A**); SUA combined with CHD and APOB had the highest AUC for predicting AF [AUC: 0.906 (95% CI: 0.888-0.923)] (**Figure 5D**), followed by SUA combined with CHD [AUC: 0.872(95% CI: 0.851–0.893); **Figure 5B**] and APOB [AUC: 0.776(95% CI: 0.750–0.802); **Figure 5C**]. In the IFG pattern, the ROC curve analysis showed that the AUCs for using SUA, CHD, and SCr to diagnose AF were 0.60, 0.80, and 0.65, respectively (**Figure 6A**); SUA combined with CHD and SCr had the highest AUC for diagnosing AF [AUC: 0.863 (95% CI: 0.820-0.907)] (**Figure 6D**), followed by SUA combined with CHD [AUC: 0.844(95% CI: 0.796–0.891); **Figure 6B**] and APOB [AUC: 0.650(95% CI: 0.587–0.714); **Figure 6C**]. In the DM pattern, the ROC curve analysis indicated that the AUCs for taking SUA, CHD, and APOB to predict AF were 0.64, 0.79, and 0.64, respectively (**Figure 7A**); SUA combined with CHD and SBP had the highest AUC for diagnosing AF [AUC: 0.858 (95% CI: 0.818-0.898)] (**Figure 7D**), followed by SUA combined with CHD [AUC: 0.849(95% CI: 0.807–0.890); **Figure 7B**] and SBP [AUC: 0.678(95% CI: 0.623–0.734); **Figure 7C**].

## Discussion

In this Chinese case-control study, we investigated the association between SUA and AF and the correlation between SUA and metabolic factors in different FBG metabolism states. We found that males and females with AF had higher SUA levels and rates of hyperuricemia than those with controls in different FBG patterns. Meanwhile, there was a significant relationship between SUA and AF, which persisted even after adjusting for all confounding factors. SUA can be an independent risk factor for AF in different FBG metabolism patterns. Furthermore, SUA in AF patients was correlated with several different metabolic factors in different FBG patterns. Moreover, SUA and CHD are the two most significant factors for predicting AF, while factors including APOB, SCr, and SBP might help to further improve the diagnostic efficiency.

Detection of AF in patients with abnormal glucose metabolism poses a diagnostic challenge. SUA is the end product of adenine and guanine metabolism in the human body (26), and its increased level may be related to cardiometabolic and cardiovascular disease development by affecting lipid and glucose metabolism (17). Hyperuricemia, defined as SUA levels higher than 7 mg/dL in men or 6 mg/dL in women (27), has been identified as a significant risk factor for AF (6, 8), but the detailed relationships and mechanisms between SUA levels and AF remain unknown. In recent years, increased SUA levels have been reported to be associated with the induction of atrial remodeling by the inflammation and oxidative damage in the pathological process of AF, thus predicting the onset of AF (28–30); this has also been supported by a mendelian randomization analysis (31) and several meta-analyses (9, 32–34). Some studies have also explored the relationship between SUA level and AF from different perspectives. An updated meta-analysis (11) reported the relationship between SUA levels and different types of AF, SUA levels were significantly different among patients with new-onset, paroxysmal, and persistent AF; in detail, patients with persistent AF had the highest level of SUA, followed by paroxysmal AF, and the lowest level was new-onset AF. Several earlier studies investigated the gender-specific association between SUA level and AF. Suzuki et al. indicated there was an independent association between SUA levels and AF in women; both Chen et al. (35) and Lin et al. (16) have also reported similar findings. However, several other studies have reported an association between SUA levels and AF risk in both sexes (29, 36, 37). A recent Chinese study (38) showed that SUA>396.5μmol/L in AF patients indicates a severe degree of atrial fibrosis, and early intervention should be considered. Evidence suggests that elevated SUA may be involved in the occurrence and development of atrial fibrosis by inducing oxidative stress and inflammation. SUA is a marker of oxidative stress, and its increased level indicates the increase of oxidative damage. Xanthine oxidoreductase promotes oxidative stress through the formation of electron radical superoxide anion, and the upregulation of xanthine oxidase activity by elevated SUA increases oxidative damage (39). In an oxidative stress state, on the one hand, the accumulation of reactive oxygen species (ROS) and the activation of inflammatory response lead to cell necrosis and endothelial dysfunction, promote atrial muscle fiber, and lead to atrial structural remodeling; on the other hand, oxidative damage changes the level of ion channels and affects the energy of atrial contraction myofibrils, leading to atrial electrical remodeling (40, 41).

The growing recognition of the strong links between diabetes, hyperuricemia, and AF has spurred our interest in unraveling their mechanistic links. To date, there is no information on the relationship between SUA levels and AF in different patterns of glucose metabolism, although an association between elevated SUA and AF in individuals with T2DM has been demonstrated. A 10-year follow-up study by Valbusa et al. (42) revealed that elevated SUA is closely related to an increased incidence of AF in patients with type 2 diabetes after adjustment for risk factors for AF. Another two small retrospective studies by Mantovani et al. (43, 44) indicated that hyperuricemia is independently correlated with an increased risk of both AF and paroxysmal AF in patients with T2DM.

To the best of our knowledge, this is the first study describing the relationship between SUA levels and AF in different FBG metabolism patterns. In this study, we observed that AF had higher SUA levels and rates of hyperuricemia in both sexes than those with controls, and SUA can be an independent risk factor for AF in different FBG patterns after adjusting for multiple confounding factors. More importantly, we explored the ROC curve model of SUA for diagnosing AF and evaluated the diagnostic performance in different FBG metabolism patterns. Herein, We know that SUA and CHD are significant predictors of AF, while APOB, SCr, SBP, and other factors may also contribute to improving the diagnostic efficiency of AF. From a clinical point of view, the role of diabetes in promoting AF has been beyond doubt. In addition to already diagnosed diabetes mellitus, increased impaired fasting glucose in prediabetes is also associated with an increased risk of future AF (45). A study has shown that a 10 mg/dL elevate in FBG was associated with an increased risk of AF (46); additional evidence has also shown that a 1 mmol/L increase in FBG was associated with a 33% increased risk of atrial fibrillation in people who did not progress to diabetes. Therefore, it is recommended to start with prediabetes and to screen regularly for AF as IFG/T2DM progresses.

Although the pathophysiological mechanism of SUA level and AF with dysglycaemia remains controversial, the influence of concomitant risk factors such as coronary heart disease, hypertension, and metabolic dysfunction on the risk of AF should be considered. The association between diabetes and AF has been suggested to be caused by a number of diabetes-related factors, such as hypertension and obesity (47). Prehypertension and IFG, the prior stages of hypertension and diabetes, are considered potential independent risk factors of AF, especially when systolic and diastolic blood pressure elevation combined with IFG significantly increases the risk of new AF (48). Particularly, elevated SUA, a regulator of glucose and lipid metabolism, suggests a mechanism of impaired metabolic homeostasis (49). Therefore, the increase of SUA is likely to drive the association between abnormal blood glucose and AF. From this standpoint, it can be speculated that with the increase in SUA levels and dysglycemia, may play an important role in the progression of inflammation and oxidative stress in AF (50). Moreover, the possibility of preventing the development of AF by modifying risk factors associated with dysglycaemia, metabolic syndrome, and sedentary lifestyle has recently been highlighted (45). On this basis, we further investigated the correlation between SUA and metabolic factors under different FBG patterns. We found that SUA was positively correlated with FBG and SCr, whereas was negatively correlated with HDL-C and APOA1 in normoglycemic patients; SUA was positively correlated with LDL-C and SCr, whereas was negatively correlated with HDL-C in the IFG pattern. Increased SUA levels may be related to decreased renal excretion (51), and promote the progression of renal injury by activating the renin-angiotensin system and destroying vascular endothelial function (52) while inducing the activation of oxidative stress and inflammatory pathways. Elevated SCr is usually indicative of renal injury, which might explain our findings.

Atherosclerosis and CHD are the key factors that trigger AF and inflammatory mechanisms. The prominent role of inflammation in the pathogenesis of atherosclerosis and its complications including AF cannot be ignored, and its inducing factors are still unclear, but lipid disorders cannot be excluded (53). Elevated SUA has also been proposed to be associated with unstable coronary plaques (54). In addition, ample evidence implicates that insulin resistance and the insulin resistance (metabolic) syndrome contribute to the increase of cardiovascular risk in patients with T2DM (55). Insulin resistance, a precursor to metabolic syndrome and DM, appears at almost any stage of atherosclerotic disease, from endothelial dysfunction to accelerated atherosclerotic progression, plaque vulnerability, and finally coronary events (56). Finally, from the consequence of the ROC curve, we recommend the use of SUA, CHD, and APOB to predict AF in normoglycemic patients; SUA, CHD, and SCr to predict AF in IFG patients; SUA, CHD, and SBP to predict AF in DM patients. Collectively, these findings are supported by previous evidence and contribute to a better understanding of the relationship between SUA, dysglycemia, and AF pathology.

Several limitations of the current study should be acknowledged. First, a major limitation of this study is its retrospective design, which, while preventing any knowledge of causality, can form a hypothesis for future studies to confirm. Second, the size of the population and the number of research centers is indeed limited; in this context, it is inevitable to increase the heterogeneity and inaccuracy. Third, in this study, SUA and FBG at a single point are not representative of the overall status, which may have affected the output of the results. Glycosylated hemoglobin (HbA1c) has not been routinely analyzed and was limited to a small population, so it is not currently included in the analysis. Fourth, we failed to focus on subgroup analyses of AF type, sex, and age, which may have missed some important information; additionally, information on the type of diabetes is also not available. Fifth, markers of inflammation and oxidative stress were not evaluated, which could have confounded the current results. Finally, we cannot exclude residual confounding factors, including body mass index (BMI), physical activity, smoking, and drinking. Meanwhile, we only focused on the population diagnosed with AF on admission, some patients had AF before admission and were ignored due to delay in treatment and unclear diagnosis, especially asymptomatic AF. Notwithstanding these limitations, our study is the first to explore the association between SUA levels and AF in different patterns of FBG metabolism and has significant strengths, including a relatively complete dataset and the ability to adjust for multiple confounding factors, while also exploring the performance of SUA levels in diagnosing AF. Importantly, it provides a new perspective to further understand the pathologic process of AF and the possibility of tightly managing SUA levels to reduce the risk of AF in dysglycemic modes.

## Conclusions

In conclusion, increased SUA and AF were independently correlated in different FBG metabolic patterns. Moreover, this relationship may be driven by several risk factors for AF, including CHD, APOB, SCr, and SBP. Elevated SUA, as a key regulator of glucose and lipid metabolism, may contribute to complex inflammation and oxidative stress in AF. The current findings offer the possibility of preventing AF in different stages of FBG metabolism to a certain extent: in the normoglycemic pattern, SUA, CHD, and APOB levels should be monitored with emphasis; in the FIG pattern, SUA, CHD, and SCr level should be monitored; in the DM pattern, attention should be paid to the levels of SUA, CHD, and SBP. The exact mechanisms of SUA participation in glycemic metabolism or the pathogenesis of AF and evaluation of SUA-lowering therapies, as well as their impact on clinical outcomes, remain to be investigated longitudinally.

## Data availability statement

The raw data supporting the conclusions of this article will be made available by the authors, without undue reservation.

## Ethics statement

Written informed consent was not obtained from the individual(s) for the publication of any potentially identifiable images or data included in this article.

## Author contributions

HJ was the main coordinator of the project and was responsible for the study design. XZ and HJ drafted the manuscript of the present paper. MY and JT were involved in the supervising of data collection and stratification. XZ and DZ contributed to data assembly and analysis. All authors contributed to the article and approved the submitted version.
